# Pregnant in a Pandemic: Connecting Perceptions of Uplifts and Hassles to Mental Health

**DOI:** 10.1177/13591053221120115

**Published:** 2022-08-29

**Authors:** Stephanie M Reich, Nestor Tulagan, Melissa Dahlin, Sina Labaff, Nikil Dutt, Amir Rahmani

**Affiliations:** University of California, Irvine, CA, USA

**Keywords:** COVID-19, hassles, loneliness, mental health, positivity, pregnancy, uplifts

## Abstract

How women experience pregnancy as uplifting or a hassle is related to their mental and physical health and birth outcomes. Pregnancy during a pandemic introduces new hassles, but may offer benefits that could affect how women perceive their pregnancy. Surveying 118 ethnically and racially diverse pregnant women, we explore (1) women’s traditional and pandemic-related pregnancy uplifts and hassles and (2) how these experiences of pregnancy relate to their feelings of loneliness, positivity, depression, and anxiety. Regressions show that women who experience more intense feelings of uplifts than hassles also feel more positive, less lonely, and have better mental health. Findings suggest that focusing on positive aspects of being pregnant, in general and during a pandemic, might be beneficial for pregnant women’s mental health.

## Introduction

Pregnant women’s mood is related not just to their mental and physical health, but to birth outcomes as well (Akiki et al., 2016; Bedaso et al., 2021; Lobel et al., 2008a). Though much attention in the extant literature has been placed on the risks that negative feelings, such as stress, anxiety, and depression, can have on pregnant women’s and their fetuses’ health (Bedaso et al., 2021; Lobel et al., 2008a; Zijlmans et al., 2017), studies are increasingly documenting how happiness, excitement, and positivity during pregnancy are related to wellbeing, smoother deliveries, and healthier newborns (Amiel Castro et al., 2020; McManus et al., 2017).

The COVID-19 pandemic has introduced new stressors for pregnant women, but may also offer benefits. With social distancing, women may feel more isolated in daily life and when seeking medical care (e.g. check-ups alone) as well as have fears related to this new disease (Kolker et al., 2021). However, women may enjoy aspects of pandemic-related policies and practices, such as spending more time at home, having easier access to bathrooms, nap spaces, and comfortable clothing, and feeling a less hectic pace to daily life. Given the importance of women’s feelings about their pregnancy and the new challenges and affordances presented by the COVID-19 pandemic, we explore how women’s feelings of pregnancy-related uplifts and hassles are associated with their feelings of loneliness, sadness, anxiety, and positivity. Importantly, we consider traditionally-measured pregnancy uplifts and hassles (DiPietro et al., 2004) and newer ones that are specific to the current pandemic.

### Negative prenatal mood, health, and child outcomes

Studies have documented how women’s feelings during pregnancy relate to their health and wellbeing. Research has consistently identified the detrimental impacts of prenatal stress, anxiety, and depression on prenatal and postnatal outcomes (Graignic-Philippe et al., 2014; Lee and Hans, 2015; Lobel et al., 2008a). Women who feel stressed, sad, or anxious during pregnancy are more likely to have shorter gestation times, preterm labor, lower infant birthweight, and worse newborn health (e.g. Accortt et al., 2015; Liou et al., 2016). In a longitudinal study of 3376 pregnant women, researchers found that women who scored one standard deviation (SD) above the mean on depression and anxiety had gestational times that were 0.04–0.05 SD units shorter than the average (Pesonen et al., 2016). Other studies have found prenatal anxiety, distress, and stress to be associated with preterm labor and lower APGAR scores at birth (Grote et al., 2010; Hasanjanzadeh and Faramarzi, 2017). Further, women who experience anxiety and depression during pregnancy are more likely to experience postpartum depression and anxiety (Heron et al., 2004), which is associated with a host of poor maternal and child outcomes (Jacques et al., 2019).

Though clinical levels of prenatal depression, anxiety, and stress are robustly tied to poor prenatal and fetal health, mounting evidence notes the risk of negative feelings, even below clinical levels. Depressive symptoms are linked to prenatal physiologic stress, fetal brain development, and child outcomes (e.g. Braithwaite et al., 2015; Lebel et al., 2016; Liou et al., 2016). For instance, women who report high stress during pregnancy are more likely to have 6-month-old infants with negative emotional reactivity (Nolvi et al., 2016) and those with depressive symptoms during pregnancy are more likely to have children who show less cortical thickness and white matter on MRI scans in preschool (Lebel et al., 2016) than those without these symptoms.

With the COVID-19 pandemic, stress and anxious and depressive symptoms appear to be higher (Hessami et al., 2020), especially for pregnant women (Lebel et al., 2020; López-Morales et al., 2021). For instance, a geocoded and age-matched comparison of pregnant women before and during the COVID-19 pandemic found that women were nearly twice as likely to experience depression during the pandemic (King et al., 2021). Additionally, with social distancing policies in place, pregnant women are also more likely to report feeling lonely (Kolker et al., 2021).

Though negative feelings during pregnancy have been extensively studied and are a robust area of pandemic-related research, far less focus has been placed on positive feelings during pregnancy, especially during the COVID-19 pandemic. The pandemic clearly introduced new and acute stressors (Wall and Dempsey, 2022), but there may also be some pandemic-related benefits, which have not yet been studied.

### Positive prenatal mood, health, and child outcomes

Though far less studied than negative prenatal feelings, positive feelings during pregnancy, including feelings about the pregnancy, birth, and transition to motherhood, are associated with a range of beneficial outcomes from better maternal mental health and fetal growth to infant and child outcomes (Amiel Castro et al., 2020; DiPietro et al., 2002; Dipietro et al., 2008; McManus et al., 2017; Yali and Lobel, 2002). Pregnant women who feel more positive and hopeful are more likely to obtain prenatal care (Hoseini et al., 2020), breastfeed after birth (McManus et al., 2017), and have infants with fewer sleep problems (Liu et al., 2020). A recent study of almost 3400 mother–child dyads found that the more positive women felt during pregnancy, the less likely their children were to have a mental or behavioral disorder diagnosis from birth to 12 years of age (Lähdepuro et al., 2022).

Given that positive feelings during pregnancy are far less studied than negative ones, it is not surprising that very little research on prenatal mental health during the pandemic has looked at the prevalence or consequences of positive prenatal feelings. One notable cross-sectional survey in Australia found that pregnant women who reported being a happy person tended to value health and be physically active each week, meeting the Australian guidelines of 150 minutes of exercise per week (Christie et al., 2021). Another cross-sectional study of 161 pregnant women found women’s meaning in life to be significantly associated with their life satisfaction and happiness during the pandemic (Majercakova Albertova and Bolekova, 2022). Though important contributions toward addressing these gaps, none of the extent pandemic-related research to our knowledge has looked at positive and negative feelings specifically related to pregnancy, or how they might relate to women’s prenatal mental health.

### Prenatal uplifts and hassles

Research has demonstrated the benefit of considering the prevalence and intensity of positive and negative feelings about pregnancy specifically and how they relate to each other (Amiel Castro et al., 2020; Faramarzi et al., 2016). Pregnancy inherently has hassles and enjoyable aspects, and women’s subjective experiences of these are related to their prenatal health, fetus and infant health, and parenting practices after birth (Amiel Castro et al., 2020; McManus et al., 2017; Verner et al., 2021). The most common measurement of these uplifts and hassles of pregnancy is the Pregnancy Experience Scale (DiPietro et al., 2004; Dipietro et al., 2008), which compares the number of uplifts in relation to hassles and the intensity of pregnancy uplifts and hassles on a Likert Scale. Research has supported both the use of the frequency and intensity ratios. For instance, women who reported more hassles than uplifts and more intense hassles than uplifts during pregnancy were significantly more likely to have a 24- to 36-month-old infant with higher arousal than sociability and more total behavior problems as measured with the Child Behavior Checklist (CBCL) (Bowers et al., 2021). In another study, women who reported more frequent and more intense uplifts than hassles were more likely to have secure attachments with their infant, and those women who reported more hassles were also more likely to rate their infant, as fussy (DiPietro et al., 2002).

Considering women’s feelings of pregnancy uplifts and hassles in conjunction with their prenatal mental health appears to have utility for understanding prenatal risk and resilience. For instance, a recent study found that women’s experiences of uplifts and hassles during pregnancy coupled with social support, anxiety, stress, and positive and negative affect predicted telomere length of DNA from cord blood samples (Verner et al., 2021), suggesting impacts of prenatal feelings on cellular aging. Further, women who focused more on the hassles and negative aspects of pregnancy tended to experience more stress and anxiety (Akiki et al., 2016; Lobel et al., 2008b) and have children with worse postnatal health outcomes (Souza-Vogler and Lima, 2021; Zijlmans et al., 2017).

Thus, negative feelings (e.g. stress, depression, anxiety) while pregnant appear to be detrimental to women and their offspring (Bedaso et al., 2021; Braithwaite et al., 2015). Conversely, positive feelings (hope, happiness, purpose) tend to be beneficial to women and children (Golmakani et al., 2012; Hoseini et al., 2020). However, feelings specific to the pregnancy, especially how the pregnancy is perceived as uplifting or a hassle have unique risks and benefits (Amiel Castro et al., 2020; McManus et al., 2017). Unfortunately, how women experience their pregnancy during the COVID-19 pandemic as uplifting or a hassle has not been well studied, and the ways in which the pandemic may introduce new hassles or benefits have not yet been investigated. Further, these pregnancy experiences during the pandemic have not been connected to women’s mental health. Therefore, we explore:

1. How do pregnant women during the COVID-19 pandemic experience:
  - traditional pregnancy uplifts and hassles?
  - pandemic-specific pregnancy uplifts and hassles?
2. How does the intensity of pregnancy uplifts to hassles, both traditional and pandemic-specific, relate to women’s feelings of positivity, loneliness, depression and anxiety?

## Method

Pregnant women were recruited for this anonymous online survey through flyers placed in obstetric offices (including perinatalogist offices), Women, Infants, and Children (WIC) clinics, and WIC nutrition centers in southern California. The flyers were in English and Spanish and provided a tiny URL and QR-code for accessing English or Spanish versions of the survey. The survey started with a study information sheet, which described the study details including risks and benefits. Respondents were informed that clicking the “continue” button meant that they consented to participate. The survey concluded with a link to another platform where respondents could enter their email address for a $10 Amazon gift card. This ensured there was no way to connect an email address with survey responses. All procedures and materials were reviewed and approved by a university Institutional Review Board.

### Participants

From October 2020 to March 2021, 118 women completed the anonymous survey exploring their experiences of being pregnant during a pandemic. The sample was 38% Latina, 38% White, 19% Asian, and 5% other/multi-ethnic. On average, participants were 30 years (range = 18–46; SD = 5.5) and in their 27th week of pregnancy, though the range was large from 8 to 41 weeks (SD = 8.6). Most (78%) were partnered and 73% of the pregnancies were planned. Approximately 46% reported not having any other children. At the time of data collection, 10% currently had or had recently recovered from COVID-19. Nearly 45% had one or more health conditions, some of which were pregnancy related, including: gestational diabetes (12%), placenta previa (1%), or another pregnancy-related health condition (e.g. preeclampsia, high risk for preterm labor) (7%). Thus, about one in five women had a pregnancy-related health problem. See Table 1 for details.

**Table 1.** Participant characteristics.

Variable	Mean/%	SD/count	Min	Max
Traditional uplifts	1.57	0.70	0.30	3.00
Traditional hassles	1.31	0.70	0.00	3.00
Traditional uplifts-to-hassles ratio	1.23	0.54	0.3	3.43
Pandemic-related uplifts	1.58	0.76	0.00	3.00
Pandemic-related hassles	1.81	0.77	0.00	3.00
Pandemic-related uplifts-to-hassles ratio	1.00	0.45	0.40	3.75
Positivity	19.63	3.64	7.00	25.00
Depression/anxiety	2.91	2.39	0.00	9.00
Loneliness	5.16	1.89	3.00	9.00
Financial strain	2.31	0.68	1.30	4.00
Race/ethnicity				
White	39%	47	0%	100%
Latina	32%	38	0%	100%
Asian	19%	22	0%	100%
Other	11%	13	0%	100%
Age (in years)	30.19	5.49	18.00	46.00
# of weeks pregnant	27.06	8.61	8.00	41.00
Respondent has a partner	78%	94	0%	100%
Pregnancy was planned	73%	86	0%	100%
Respondent has no other children	46%	54	0%	100%
# of children total	0.88	1.15	0.00	5.00
Health conditions				
COVID-19 exposure	10%	12	0%	100%
Pregnancy-related health condition	20%	23	0%	100%
Health condition	45%	53	0%	100%

*N* = 118.

### Anonymous survey

Women were first asked about their background characteristics and financial strain related to the pandemic, then about their pregnancy experiences of uplifts and hassles followed by feelings of loneliness, positivity, sadness and anxiety.

#### Pregnancy uplifts and hassles

The 20-item Pregnancy Experience Scale (PES), uses a 4-point Likert scale to measure the intensity of traditional uplifts (10 items) and hassles (10 items) during pregnancy (Dipietro et al., 2008). We believe the widespread use of social media is an important conduit for sharing pregnancy information and connecting to others; thus, we added one additional uplift to capture sharing about pregnancy on social media. For this paper, we term these 21 items as traditional uplifts and hassles. Along with these traditional items, pregnant women also answered eight new uplifts and 12 new hassles items related to the COVID-19 pandemic. Confirmatory factor analyses (CFA) dividing these items into four factors of traditional uplifts, traditional hassles, pandemic-related uplifts, and pandemic-related hassles demonstrated good model fit, χ^2^(239) = 331.195, *p* < 0.001, RMSEA [90% CI] = 0.06 [0.04–0.07], CFI/TLI = 0.917/0.904 (see Supplemental Table S1 CFA results).

The traditional uplifts factor consisted of nine items encompassing aspects of pregnancy that make women feel happy, positive, or uplifted (e.g. discussion about baby’s name and pregnancy; Cronbach’s α: 0.86; CFA loadings: 0.54–0.73). Two of the 11 traditional uplift items were omitted due to low factor loadings (see Supplemental Table S2 for details). The traditional hassles consisted of six items involving aspects that make the women feel negative, upset, or unhappy (e.g. discomforts of pregnancy, clothes not fitting; Cronbach’s α: 0.83; CFA loadings: 0.50–0.89). Four of the 10 traditional hassle items were omitted due to low factor loadings (see Supplemental Table S2 for details). The pandemic-related uplifts consisted of four items about positive pregnancy experiences during the pandemic (e.g. convenience of sleeping and going to the bathroom easily at home, receiving unexpected gifts or cards, and having time to prepare for their baby’s arrival (Cronbach’s α: 0.80; CFA loadings: 0.54–0.88). The other four pandemic-related uplifts items were omitted due to low factor loadings (see Supplemental Table S2 for details). The pandemic-related hassles included five items about the women’s negative feelings during the pandemic (e.g. COVID-19 affecting themselves or the baby, feelings of isolation, and missing baby-related events with loved ones; Cronbach’s α: 0.80; CFA loadings: 0.54–0.88). Seven of the 12 pandemic-related hassle items were omitted due to low factor loadings (see Supplemental Table S2 for details).

For our analyses, we created average intensity scores (i.e. mean-composite scores to account for different numbers of items in subscales) for each of the four factors and then used them to create uplifts-to-hassles ratios. Traditional uplifts-to-hassles ratio divided women’s average traditional uplifts scores by their average traditional hassles scores. Pandemic-related uplifts scores were divided by pandemic-related hassles scores to create a pandemic-related uplifts-to-hassles ratio. In our analyses, ratio scores higher than 1.00 indicated higher levels of uplifts relative to hassles, and scores lower than 1.00 indicated higher levels of hassles than uplifts.

#### Positivity

Feelings of positivity were measured by the 6-item Positivity Scale (Caprara et al., 2012), in which respondents indicated their agreement on a 5-point Likert scale (*strongly disagree* = 1 to *strongly agree* = 5) to create a total summary score (with one item reverse coded for consistency) ranging from 6.0 to 30.0.

#### Loneliness

The Short Loneliness Scale was used to measure women’s feelings of loneliness (Hughes et al., 2004). This 3-item measure asked respondents to indicate the extent to which they feel a lack of companionship, left out, and isolated on a scale from *hardly ever* (1) to *often* (3). Responses were summed to create an overall loneliness score, creating a range from 3.0 to 9.0.

#### Depression and anxiety

Women were asked about their feelings of depression and anxiety with three items from the PHQ-4 (Kroenke et al., 2009). The questions asked how often women were bothered over the past 2 weeks by (1) not being able to stop or control worrying, (2) feeling down, depressed, or hopeless, and (3) having little interest or pleasure in doing things. All three items were rated on a 4-point Likert scale (0 = never to 3 = very *often*). The 3-item version has been used by others (He et al., 2021; Reich et al., 2021) and a summary score was created ranging from 0 to 12.0 (Löwe et al., 2010).

#### Financial strain and background characteristics

Pandemic-related changes in employment and ability to make ends meet were asked: “Since the COVID-19 crisis began, has your employment changed?” Responses included: “*No change*” and “*Got new job/Gained hours*,” which were scored as 0 and “*Lost job/Lost hours*” was scored as 1. Women were also asked, “Since the COVID-19 crisis began, has your ability to (1) pay your bills (e.g. rent, utilities) and (2) buy basic needs (e.g. food, diapers) changed?” Response options included: “*No change*,” “*Yes, it is easier than before*,” “*Yes, it is slightly more difficult*,” and “*Yes, it is much more difficult*.” The two categories indicating more difficulty were scored as 1 and the other two were scored as 0. These three items were summed into a financial strain variable (range: 0–3).

Women were also asked about their race/ethnicity, age, the number of weeks of their pregnancy, partner status, if the pregnancy was planned, if they had other children, if they currently had or had recently recovered from COVID-19, and if they had general (e.g. diabetes, hypertension) or pregnancy-related (e.g. gestational diabetes, preeclampsia) health problems.

### Analytic plan

To answer research questions 1a and 1b, we estimated the statistical means of each factor of traditional and pandemic-related uplifts and hassles and the correlations across these factors using Stata 14.2. To answer research question 2, we conducted sequential multiple regression analyses to estimate the unique contributions of traditional and pandemic-related uplifts-to-hassles ratios on pregnant women’s psychological wellbeing (i.e. positivity, depression/anxiety, and loneliness) in separate models. Given the unique benefits of positivity and its lower prevalence in the literature, we opted for separate models, rather than create a general wellbeing composite. In the first step, we tested associations between the traditional uplifts-to-hassles ratio and a given outcome. We accounted for financial strain and background characteristics like race/ethnicity, age, weeks of gestation, partner status, having other children, if the pregnancy was planned, COVID-19 exposure, and if the women had pregnancy-related and general health issues. Then, in the second step, we added the pandemic-related uplifts-to-hassles ratio as a predictor to examine its unique contribution to each outcome.

To account for our relatively modest sample size and to achieve parsimony, we omitted background covariates that did not predict any of the outcomes at the *p* < 0.10 level and re-estimated the final models. Only financial strain, race/ethnicity, partner status, and pregnancy-related health issues predicted at least one outcome and were thus retained in the final models. Women’s age, weeks of gestation, if the pregnancy was planned, if women had other children, COVID-19 exposure, and general health issues did not significantly predict any of the outcomes and were thus dropped from the final models.

## Results

### Preliminary statistics

In this sample, women had moderately high positivity (*M* = 19.63, SD = 3.64; range: 7–25), low depression/anxiety (*M* = 2.91, SD = 2.39; range: 0–9), and moderate loneliness (*M* = 5.16, SD = 1.89; range: 3–9). See Table 1 for details. Additionally, women’s positivity was negatively correlated with both depression/anxiety (*r =* −0.33 to −0.46, *p* < 0.001) and loneliness (*r =* −0.33 to −0.46, *p* < 0.001), and depression/anxiety was positively correlated with loneliness (*r =* −0.33 to −0.46, *p* < 0.001). Finally, both the traditional and pandemic-related uplifts-to-hassles ratios were also positively correlated with positivity (*r*’s* =* 0.38, *p* < 0.001) and negatively correlated with depression/anxiety (*r* *=* −0.33 to −0.46, *p* < 0.001) and loneliness (*r =* −0.33 to −0.45, *p* < 0.001). See Table 2.

**Table 2.** Correlations among main study variables.

Variables	1	2	3	4	5
1. Traditional uplifts-to-hassles ratio	1.00				
2. Pandemic-related uplifts-to-hassles ratio	0.32***	1.00			
3. Positivity	0.38***	0.38***	1.00		
4. Depression	−0.46***	−0.33***	−0.49***	1.00	
5. Loneliness	−0.45***	−0.33***	−0.46***	0.57***	1.00

*N* = 118.

*** *p* < 0.001.

### Intensity of traditional and pandemic-related uplifts and hassles

Our first research question examined how pregnant women experienced traditional and pandemic-related uplifts and hassles during the COVID-19 pandemic. Descriptive and correlational statistics are presented in Tables 1 and 2. With regard to traditional uplifts and hassles, on average, women experienced moderate levels of traditional uplifts (*M* = 1.57, SD = 0.70; range: 0.33–3) and traditional hassles (*M* = 1.31, SD = 0.70; range: 0–3) during the COVID-19 pandemic and slightly higher levels of traditional uplifts relative to traditional hassles (ratio *M* = 1.23, SD = 0.54; range: 0.33–3.43).

Women also experienced moderate levels of pandemic-related uplifts (*M* = 1.63, SD = 0.83; range: 0–3) and moderately high levels of pandemic-related hassles (*M* = 1.81, SD = 0.77; range: 0–3) on average. Women also had relatively equal levels of pandemic-related uplifts to pandemic-related hassles on average (ratio *M* = 1.00, SD = 0.45; range: 0.39–3.75). Lastly, the traditional uplifts-to-hassles ratio was positively correlated with the pandemic-related uplifts-to-hassles ratio (*r =*0.32, *p* = 0.004).

### Associations between uplifts-to-hassles ratios and psychological wellbeing

Our second research question tested the unique contributions of traditional and pandemic-related uplifts-to-hassles ratios on women’s positivity, depression/anxiety, and loneliness. We present results from our multiple regression analyses in Table 3.

**Table 3.** Regressions using pregnancy uplifts and hassles to predict women’s mental health.

Predictors	Positivity		Depression		Loneliness	
	*B* (SE)	*B* (SE)	*B* (SE)	*B* (SE)	*B* (SE)	*B* (SE)
	*β*	*β*	*β*	*β*	*β*	*β*
Traditional uplifts-to-hassles ratio	2.68*** (0.60)	2.14*** (0.60)	−2.05*** (0.37)	−1.81*** (0.38)	−1.46*** (0.29)	−1.25*** (0.29)
	0.40	0.32	−0.46	−0.41	−0.42	−0.36
Pandemic uplifts-to-hassles ratio		2.22** (0.71)		−1.01* (0.44)		−0.87* (0.34)
		0.28		−0.19		−0.21
Financial strain	0.36 (0.50)	0.46 (0.49)	0.53^†^ (0.31)	0.48 (0.30)	0.54* (0.24)	0.50* (0.23)
	0.07	0.09	0.15	0.14	0.19	0.18
Race/ethnicity (REF: White)						
Latina	−0.44 (0.76)	−0.37 (0.73)	0.55 (0.47)	0.52 (0.46)	−0.58 (0.36)	−0.61^†^ (0.35)
	−0.06	−0.05	0.11	0.10	−0.14	−0.15
Asian	0.26 (0.88)	0.60 (0.86)	0.35 (0.54)	0.20 (0.53)	−0.26 (0.42)	−0.39 (0.41)
	0.03	0.06	0.06	0.03	−0.05	−0.08
Other	1.51 (1.09)	1.36 (1.05)	−0.28 (0.66)	−0.21 (0.65)	0.26 (0.52)	0.31 (0.50)
	0.13	0.12	−0.04	−0.03	0.04	0.05
Has a partner	1.56^†^ (0.79)	1.50^†^ (0.76)	−0.79 (0.49)	−0.77 (0.48)	−1.01** (0.38)	−0.99** (0.37)
	0.18	0.17	−0.14	−0.13	−0.22	−0.22
Has pregnancy-related health issue	−0.34 (0.83)	−0.18 (0.80)	−0.87^†^ (0.51)	−0.94^†^ (0.50)	−0.16 (0.40)	−0.22 (0.39)
	−0.04	−0.02	−0.14	−0.15	−0.03	−0.04
Constant	14.28*** (1.78)	12.41*** (1.82)	4.77*** (1.09)	5.62*** (1.13)	6.73*** (0.85)	7.47*** (0.88)
*N*	118	118	118	118	118	118
*R* ^2^	0.20	0.27	0.31	0.34	0.33	0.37
Δ*R*^2^		0.07**		0.03*		0.04*

*N* = 118. Final regression model omitting covariates that did not significantly predict any of the outcomes including: women’s age, weeks of gestation, if the pregnancy was planned, if new to motherhood (no other children), COVID-19 exposure, and general health issues.

† *p* < 0.10 *p < 0.05 **p < 0.01. ***p < 0.001.

#### Positivity

In the first model, women who experienced higher levels of traditional uplifts relative to traditional hassles were more likely to feel more positive than those with lower traditional uplifts-to-hassle ratios (*B* = 2.68, SE = 0.60, *β* = 0.40, *p* < 0.001), accounting for background characteristics. This model accounted for about 20% of the variance in women’s positivity. Adding the pandemic-related uplifts-to-hassles ratio as a predictor in the second model further explained 27% of the variance in this outcome. In the second model, the significant unique effect of the traditional uplifts-to-hassles ratio on women’s positivity was retained (*B* = 2.14, SE = 0.60, *β* = 0.32, *p* < 0.001). Moreover, women with higher pandemic-related uplifts than hassles were also more likely to feel positive compared to those with lower pandemic-related uplifts-to-hassles ratios (*B* = 2.22, SE = 0.71, *β* = 0.28, *p* = 0.002), above the effects of the traditional uplifts-to-hassles ratio and background characteristics.

#### Depression/anxiety

Accounting for background characteristics, women who had higher traditional uplifts than hassles were less likely to feel depression/anxiety (*B* = −2.05, SE = 0.37, *β* = −0.46, *p* < 0.001) than those with lower traditional uplifts-to-hassles ratios. This model accounted for 31% of the variance in women’s depression/anxiety. The significant effects of traditional uplifts-to-hassles ratio remained after adding pandemic-related uplifts-to-hassles ratios in the second modeling step (*B* = −1.81, SE = 0.38, *β* = −0.41, *p* < 0.001). Additionally, women who experienced higher levels of pandemic-related uplifts than hassles felt less depressed/anxious compared to those who experienced lower pandemic-related uplifts-to-hassles ratios (*B* = −1.01, SE = 0.44, *β* = −0.19, *p* = 0.025). This second model also explained 34% in the variance in this outcome.

#### Loneliness

Finally, with regard to women’s feelings of loneliness, women with higher levels of traditional uplifts than hassles were less likely to experience loneliness compared to those with lower traditional uplifts-to-hassles ratios (*B* = −1.46, SE = 0.29, *β* = −0.42, *p* < 0.001). This model accounted for about 33% of the variance in women’s feelings of loneliness. Adding pandemic-related uplifts-to-hassles ratios as a predictor in the second model further explained 37% of the variance in this outcome (Δ*R*² = 0.04, *p* = 0.012). The significant unique effect of the traditional uplifts-to-hassles ratio on women’s loneliness was retained in this second modeling step (*B* = −1.25, SE = 0.29, *β* = −0.36, *p* < 0.001). Women who experienced higher pandemic-related uplifts than hassles were also less likely to feel lonely than those with lower pandemic-related uplifts-to-hassles ratios (*B* = −0.87, SE = 0.34, *β* = −0.21, *p* = 0.012), above the effects of the traditional uplifts-to-hassles ratio and background characteristics.

## Discussion

How women perceived their pregnancy as uplifting or a hassle was related to their mental health, replicating other studies in which greater intensity of hassles is associated with more stress, anxiety, and worse coping (Faramarzi et al., 2016; Voegtline et al., 2013). Pregnancy hassles and uplifts specifically related to the COVID-19 pandemic were also significantly associated with women’s mental health, though the ratio of pandemic-related uplifts-to-hassles was less predictive of women’s feelings of depression/anxiety, loneliness, and positivity than the traditional ratio. Nonetheless, including both types of ratios accounted for up to 37% of the variance of women’s positive and negative (loneliness, depression/anxiety) feelings. Such findings have practical significance, as helping women focus on the good parts of pregnancy, even those that are unique to the pandemic, might be an effective way to address anxiety, depression, and feelings of loneliness—and promote more positivity. Though these data are not causal, the covariation between pregnancy experiences and mental health are important and offer insights into potential avenues for intervention. As the only study to our knowledge to consider both the hassles and uplifts of being pregnant during a pandemic, these data indicate that consideration of positive and negative experiences related to this sociohistorical time period are important.

### Importance of prenatal mental health

Prenatal stress, loneliness, sadness, and anxiety are detrimental to prenatal and postnatal outcomes (Graignic-Philippe et al., 2014; Lee and Hans, 2015; Lobel et al., 2008a). Recent meta-analyses of anxiety, stress, and depression have found higher rates of each during this pandemic (Arora et al., 2022; Hessami et al., 2020), especially among pregnant women (Demissie and Bitew, 2021; López-Morales et al., 2021). Loneliness is also detrimental to women’s and children’s health and is often co-occurring with prenatal depression (Luoma et al., 2019). Nascent survey research has found that pregnant women frequently report feelings of loneliness during social distancing policies (Giurgescu et al., 2022; Kolker et al., 2021).

Decades of research have documented the myriad ways poor prenatal mental health impacts women and fetal development (Graignic-Philippe et al., 2014; McManus et al., 2017; Zijlmans et al., 2017). Further, poor prenatal mental health is predictive of poor postnatal mental health (Heron et al., 2004; Huizink et al., 2017; Zelkowitz et al., 2008), sub-optimal parenting practices (Pearson et al., 2012), and poor infant and child outcomes (Jacques et al., 2019; Lebel et al., 2016; Zijlmans et al., 2017). Thus, our findings of significant relationships between women’s feelings about their pregnancy experience and prenatal mental health are important. Whether causal or simply an indicator of mental health risks, assessing how women perceive their pregnancy to be uplifting or bothersome is important for identifying, or perhaps intervening to reduce, feelings of anxiety, depression, and loneliness.

### Positivity and pregnancy enjoyment

Research focused on positive and uplifting aspects of pregnancy finds benefits to maternal wellbeing (Amiel Castro et al., 2020; Faramarzi et al., 2016) as well as beneficial parenting practices and positive child health outcomes (McManus et al., 2017). For instance, women’s self-reported happiness and positivity during pregnancy are positively related to coping with labor pains (Golmakani et al., 2012), higher infant birth weight (Keeley et al., 2004), and postnatal infant-maternal synchronicity (Moore et al., 2016). Though far less well studied than negative feelings, positive feelings appear to be beneficial for fetal and infant development and potentially protective against prenatal and postnatal depression, stress, and anxiety (Grote and Bledsoe, 2007; Lobel et al., 2002).

Most research focuses on the stressors of pregnancy, but our data suggest that focusing on positive aspects of being pregnant, especially during this unique time in history, might be beneficial. The lack of differences found for women with or without other children indicates the importance of uplifting feelings about pregnancy, regardless of whether women are transitioning into motherhood or already have parenting experience. The COVID-19 pandemic introduced new stressors for pregnant women (Kolker et al., 2021; Wall and Dempsey, 2022), but our data suggest that there might also be some pandemic-related benefits, which, to our knowledge, have not been studied previously. In our sample, women who focused more on these pandemic-related uplifts than hassles were more positive, less lonely, and less depressed/anxious.

### COVID-19, financial strain, and pregnancy

Importantly, many (10%) of these women currently had or had just recovered from COVID-19. Additionally, many were dealing with health issues related to pregnancy (20%) and other causes (35%). Thus, this was a relatively higher risk sample of pregnant women. However, physical health was not significantly related to women’s perceptions of their pregnancy as uplifting or a hassle nor women’s mental health. Of all the covariates included, pandemic-related financial strain was significantly associated with feelings of loneliness. This could be related to loss or reduction of employment (and subsequently, coworker contact), reduced time for socializing when working more to make ends meet, or global feelings of struggling alone to meet financial needs. Others have also found financial strain during this pandemic to be related to feelings of loneliness (Stevenson and Wakefield, 2021). However, having a partner was associated with fewer feelings of loneliness. Thus, relationship status was may play a compensatory role, as women with partners felt less lonely despite the negative effects of financial strain. Future research should explore the ways in which financial strain may be related to loneliness, especially for those who do not have partners to coparent with.

### Limitations

As an anonymous online survey, this study has some inherent limitations. First, we cannot confirm the identity of respondents, though recruiting exclusively in spaces that serve pregnant women increases the likelihood that pregnant women were the respondents. Second, our recruitment through physical spaces likely attracted respondents who felt well enough to leave the house. Women with very poor mental health might have been less likely to participate. Third, women’s pregnancy spanned from the first through the third trimester. Though women’s entire pregnancies completely spanned the pandemic, we are not able to compare how pregnancy experiences might have differed at different points of pregnancy and SARS-CoV-2 positivity rates. Fourth, as a short survey, we limited the number of questions we asked and therefore are missing some demographic details about participants, such as employment or miscarriage history. Fifth, as an anonymous survey, we opted not to ask about COVID-19-related loss, as we felt the risk was greater than the benefit when raising the topic of grief without a researcher present to help or support the respondent. Sixth, women were recruited in California and their feelings may not generalize to women in other regions. Finally, and most importantly, these data are cross-sectional and cannot speak to causality. However, patterns of covariation are important for informing future experimental work, even if directionality cannot be determined.

## Conclusions

How women balance their perceptions of the positive and negative aspects of pregnancy is related to their mental and physical health. Being pregnant during the COVID-19 pandemic introduced new potential hassles and benefits. Considering how women feel about their new and traditional uplifts and hassles appears to be important and offers insights for practitioners to consider ways to make the benefits more salient, such as discussions of things women like about being pregnant, especially during a pandemic. Perhaps focusing on the positive aspects of pregnancy, from thinking about baby names to having time to prepare for the arrival under social distancing policies, might help outweigh the hassles and stressors. Such framing might support women in being more resilient during this challenging time and may contribute to better prenatal wellbeing.

## Research Data

sj-dat-5-hpq-10.1177_13591053221120115 – for Pregnant in a Pandemic: Connecting Perceptions of Uplifts and Hassles to Mental Health Click here for additional data file. sj-dat-5-hpq-10.1177_13591053221120115 for Pregnant in a Pandemic: Connecting Perceptions of Uplifts and Hassles to Mental Health by Stephanie M Reich, Nestor Tulagan, Melissa Dahlin, Sina Labaff, Nikil Dutt and Amir Rahmani in Journal of Health Psychology This article is distributed under the terms of the Creative
Commons Attribution 4.0 License ( http://www.creativecommons.org/licenses/by/4.0/) which
permits any use, reproduction and distribution of the work without
further permission provided the original work is attributed as specified
on the SAGE and Open Access pages ( https://us.sagepub.com/en-us/nam/open-access-at-sage).

sj-do-3-hpq-10.1177_13591053221120115 – for Pregnant in a Pandemic: Connecting Perceptions of Uplifts and Hassles to Mental Health Click here for additional data file. sj-do-3-hpq-10.1177_13591053221120115 for Pregnant in a Pandemic: Connecting Perceptions of Uplifts and Hassles to Mental Health by Stephanie M Reich, Nestor Tulagan, Melissa Dahlin, Sina Labaff, Nikil Dutt and Amir Rahmani in Journal of Health Psychology This article is distributed under the terms of the Creative
Commons Attribution 4.0 License ( http://www.creativecommons.org/licenses/by/4.0/) which
permits any use, reproduction and distribution of the work without
further permission provided the original work is attributed as specified
on the SAGE and Open Access pages ( https://us.sagepub.com/en-us/nam/open-access-at-sage).

sj-docx-1-hpq-10.1177_13591053221120115 – for Pregnant in a Pandemic: Connecting Perceptions of Uplifts and Hassles to Mental Health Click here for additional data file. sj-docx-1-hpq-10.1177_13591053221120115 for Pregnant in a Pandemic: Connecting Perceptions of Uplifts and Hassles to Mental Health by Stephanie M Reich, Nestor Tulagan, Melissa Dahlin, Sina Labaff, Nikil Dutt and Amir Rahmani in Journal of Health Psychology This article is distributed under the terms of the Creative
Commons Attribution 4.0 License ( http://www.creativecommons.org/licenses/by/4.0/) which
permits any use, reproduction and distribution of the work without
further permission provided the original work is attributed as specified
on the SAGE and Open Access pages ( https://us.sagepub.com/en-us/nam/open-access-at-sage).

sj-docx-8-hpq-10.1177_13591053221120115 – Supplemental material for Pregnant in a Pandemic: Connecting Perceptions of Uplifts and Hassles to Mental Health Click here for additional data file. Supplemental material, sj-docx-8-hpq-10.1177_13591053221120115 for Pregnant in a Pandemic: Connecting Perceptions of Uplifts and Hassles to Mental Health by Stephanie M Reich, Nestor Tulagan, Melissa Dahlin, Sina Labaff, Nikil Dutt and Amir Rahmani in Journal of Health Psychology

sj-dta-4-hpq-10.1177_13591053221120115 – for Pregnant in a Pandemic: Connecting Perceptions of Uplifts and Hassles to Mental Health Click here for additional data file. sj-dta-4-hpq-10.1177_13591053221120115 for Pregnant in a Pandemic: Connecting Perceptions of Uplifts and Hassles to Mental Health by Stephanie M Reich, Nestor Tulagan, Melissa Dahlin, Sina Labaff, Nikil Dutt and Amir Rahmani in Journal of Health Psychology This article is distributed under the terms of the Creative
Commons Attribution 4.0 License ( http://www.creativecommons.org/licenses/by/4.0/) which
permits any use, reproduction and distribution of the work without
further permission provided the original work is attributed as specified
on the SAGE and Open Access pages ( https://us.sagepub.com/en-us/nam/open-access-at-sage).

sj-inp-6-hpq-10.1177_13591053221120115 – for Pregnant in a Pandemic: Connecting Perceptions of Uplifts and Hassles to Mental Health Click here for additional data file. sj-inp-6-hpq-10.1177_13591053221120115 for Pregnant in a Pandemic: Connecting Perceptions of Uplifts and Hassles to Mental Health by Stephanie M Reich, Nestor Tulagan, Melissa Dahlin, Sina Labaff, Nikil Dutt and Amir Rahmani in Journal of Health Psychology This article is distributed under the terms of the Creative
Commons Attribution 4.0 License ( http://www.creativecommons.org/licenses/by/4.0/) which
permits any use, reproduction and distribution of the work without
further permission provided the original work is attributed as specified
on the SAGE and Open Access pages ( https://us.sagepub.com/en-us/nam/open-access-at-sage).

sj-log-2-hpq-10.1177_13591053221120115 – for Pregnant in a Pandemic: Connecting Perceptions of Uplifts and Hassles to Mental Health Click here for additional data file. sj-log-2-hpq-10.1177_13591053221120115 for Pregnant in a Pandemic: Connecting Perceptions of Uplifts and Hassles to Mental Health by Stephanie M Reich, Nestor Tulagan, Melissa Dahlin, Sina Labaff, Nikil Dutt and Amir Rahmani in Journal of Health Psychology This article is distributed under the terms of the Creative
Commons Attribution 4.0 License ( http://www.creativecommons.org/licenses/by/4.0/) which
permits any use, reproduction and distribution of the work without
further permission provided the original work is attributed as specified
on the SAGE and Open Access pages ( https://us.sagepub.com/en-us/nam/open-access-at-sage).

sj-out-7-hpq-10.1177_13591053221120115 – for Pregnant in a Pandemic: Connecting Perceptions of Uplifts and Hassles to Mental Health Click here for additional data file. sj-out-7-hpq-10.1177_13591053221120115 for Pregnant in a Pandemic: Connecting Perceptions of Uplifts and Hassles to Mental Health by Stephanie M Reich, Nestor Tulagan, Melissa Dahlin, Sina Labaff, Nikil Dutt and Amir Rahmani in Journal of Health Psychology This article is distributed under the terms of the Creative
Commons Attribution 4.0 License ( http://www.creativecommons.org/licenses/by/4.0/) which
permits any use, reproduction and distribution of the work without
further permission provided the original work is attributed as specified
on the SAGE and Open Access pages ( https://us.sagepub.com/en-us/nam/open-access-at-sage).
