# Supplementary material for: Pregnant in a Pandemic: Connecting Perceptions of Uplifts and Hassles to Mental Health
Source: J Health Psychol. 2022 Aug 29;28(8):711–25. doi: 10.1177/13591053221120115 (PMC10280125; doi:10.1177/13591053221120115)
Supplement: sj-docx-1-hpq-10.1177_13591053221120115 – for Pregnant in a Pandemic: Connecting Perceptions of Uplifts and Hassles to Mental Health [file sj-docx-1-hpq-10.1177_13591053221120115.docx]

**List and Description of Analysis Programs and Files**

**Stata 14 (Program used for data preparation and primary analyses)**

**Data file:**

- Name: pregpandemic.dta

**Syntax (Stata dofile)**

- Name: Pregnancy in a Pandemic_FINAL-02182022.do

**Log/Output:**

- Name: PIAP_Final.log

**Mplus 8.3 (Program used for confirmatory factor analysis)**

**Data file:**

- Name: PREG-UpliftHassle-CFA-Feb182022.dat

**Syntax (Stata dofile)**

- Name: PREG-UpliftHassle-CFA-UP and HAS-Overall-02182022.inp

**Log/Output:**

- Name: PREG-UpliftHassle-CFA-UP and HAS-Overall-02182022.out
