# Supplementary material for: Pregnant in a Pandemic: Connecting Perceptions of Uplifts and Hassles to Mental Health
Source: J Health Psychol. 2022 Aug 29;28(8):711–25. doi: 10.1177/13591053221120115 (PMC10280125; doi:10.1177/13591053221120115)
Supplement: sj-docx-8-hpq-10.1177_13591053221120115 – Supplemental material for Pregnant in a Pandemic: Connecting Perceptions of Uplifts and Hassles to Mental Health [file sj-docx-8-hpq-10.1177_13591053221120115.docx]

**Appendix**

**Table S1**

*Final Results from Confirmatory Factor Analyses on Traditional and COVID-19 Uplifts and Hassles*

| Item | Traditional Uplifts | Traditional Hassles | COVID-19 Uplifts | COVID-19 Hassles |
| --- | --- | --- | --- | --- |
| 1. Discussions about baby names | 0.73 |  |  |  |
| 1. Comments about your pregnancy/appearance | 0.56 |  |  |  |
| 1. Making/thinking about nursery arrangements | 0.65 |  |  |  |
| 1. Visits to obstetrician/midwife | 0.62 |  |  |  |
| 1. Spiritual feelings about being pregnant | 0.65 |  |  |  |
| 1. Courtesy/assistance from others b/c you are pregnant | 0.67 |  |  |  |
| 1. Thinking about the baby's appearance | 0.56 |  |  |  |
| 1. Discussions about pregnancy/childbirth issues | 0.54 |  |  |  |
| 1. Discussions about your pregnancy on social media | 0.63 |  |  |  |
| 1. Normal discomforts of pregnancy (heartburn, incontinence) |  | 0.50 |  |  |
| 1. Your weight |  | 0.80 |  |  |
| 1. Body changes due to pregnancy |  | 0.89 |  |  |
| 1. Thinking about your labor and delivery |  | 0.54 |  |  |
| 1. Ability to do physical tasks |  | 0.62 |  |  |
| 1. Clothes/shoes don't fit |  | 0.53 |  |  |
| 1. Enjoying being able to sleep when I am tired b/c at home |  |  | 0.71 |  |
| 1. Enjoying unexpected gifts or cards sent to me |  |  | 0.54 |  |
| 1. Liking always having a bathroom nearby |  |  | 0.71 |  |
| 1. Enjoying having time to prepare for the baby's arrival |  |  | 0.88 |  |
| 1. Missing out others see my growing belly/progressing pregnancy |  |  |  | 0.72 |
| 1. Worrying about how COVID-19 might affect my baby |  |  |  | 0.54 |
| 1. Missing out on baby showers/baby-related shopping |  |  |  | 0.86 |
| 1. Worrying about COVID-19 exposure when going to the doctor |  |  |  | 0.60 |
| 1. Pregnancy during the COVID-19 crisis is lonely |  |  |  | 0.63 |

*Notes.* X2 (239) = 331.195, *p* < 0.001, RMSEA [90% CI] = 0.06 [.04-.07], CFI/TLI = .917/.904.

**Table S2**

*Items Omitted from Analyses due to Low Factor Loadings in Confirmatory Factor Analyses*

| 1. Enjoying the slower pace of life during social distancing |
| --- |
| 1. Being home more gives time to prepare for the baby |
| 1. Pregnancy during C-19 crisis made pregnancy easier |
| 1. Disappointing only allowed 1 person in the delivery room with me |
| 1. Disappointed people cannot visit when baby is born |
| 1. Longing to be physically near my friends/family |
| 1. Enjoying having more time w family during this crisis |
| 1. Would like a pregnant women's group (online/social media) |
| 1. Disappointing not to have someone w me during ultrasounds |
| 1. My living arrangement is not safe |
| 1. Wish had more pregnant friends to talk with |
| 1. Amount of sleep |
| 1. Physical intimacy |
| 1. Thoughts about whether the baby is normal |
| 1. Concerns about physical symptoms (pain, spotting, etc.) |
| 1. How much is the baby moving |
| 1. Feelings about being pregnant at this time |
